# Chicago Medical Society

**Published:** 1886-05

**Authors:** 


*Stated Meeting, March 15th, 1886.*—THE PRESIDENT, C. T. PARKES, M. D., in the chair.

DR. ROBERT H. BABCOCK made remarks on

TWO CASES OF MITRAL STENOSIS, with presentation of the patients.

DR. BABCOCK said that he did not intend to give any discussion of mitral stenosis, but merely desired to present a few points of interest in connection with these patients. Mitral obstruction is due to a fusing together of the valves so that they project into the ventricle in the form of a funnel, which, according to many authors, is the most frequent form of stenosis; according to others, the obstruction is due to a septum-like valve stretching across the opening, and called the diaphragmatic valve. Owing to the obstruction to the flow of blood from the auricle into the ventricle a murmur is produced, which is rolling or blubbering in character, and occurring during the auricular systole and previous to the systole of the ventricle, is called pre-systolic or auriculo-systolic. This murmur has been graphically represented by Balfour as in some cases resembling the sound of voot-rrrb, the final t or b being the sudden, abrupt first sound produced by the ventricular systole.

In some cases no pre-systolic murmur, correctly speaking, is heard, only a diastolic murmur, which, being loudest at the apex in the mitral area, is a mitral-diastolic murmur. This murmur should be differentiated from the diastolic murmur of aortic regurgitation, which, in certain rare cases described by Balthazar Foster, has its maximum of intensity in the mitral area and not at the centre of the sternum.

In the two cases Dr. Babcock presented the murmurs differed from each other in character, in the one case the murmur being very distinctly represented by the letters voot, in the other by rrrb. The doctor was disappointed at the last moment by his inability to present a third patient, a man in whom the stenosis was indicated by a mitral-diastolic murmur which followed an impure first sound of heart, and both the impure first sound and the diastolic murmur were in this case audible at the lower angle of the left scapula. The propagation of the diastolic murmur so far to the left is very unusual; indeed, the production of the mitral-diastolic murmur is itself very rare, and is probably due to the fact that a rush of blood from the left auricle to the ventricle occurs with greater force at the beginning of the ventricular diastole than during the auricular systole, the auricle being dilated rather than hypertrophied, according to Sanson. The cases were then examined by members of the Society.

PROFESSOR E. C. DUDLEY reported a

UNIQUE CASE OF VESICO-VAGINAL FISTULA, in which the entire vesico-vaginal septum, the vaginal portion of the cervix, and anterior wall of the cervix to the internal os had sloughed away, leaving no bladder tissue between the inner extremities of the urethra and the points at which the vesico-uterine ligaments connect the bladder with the uterus. The only operation which seemed possible was to unite the posterior wall of the cervix uteri with the neck of the bladder. This would turn the uterus into the bladder and necessitate menstruation through the urethra. The anterior wall of the uterus could not be approximated to the neck of the bladder, but it was found, on further examination, that the mucous membrane of the bladder, if caught with the tenaculum about an inch in front of the uterus, could be drawn to the neck of the bladder and held without undue traction. The operator therefore undertook to close the fistula in this way by denuding a strip of the mucous membrane of the bladder from side to side an inch in front of the uterus, and thus he utilized that portion of the bladder between the line of denudation and the uterus, and made it a substitute for the lost anterior wall of the cervix and vesico-vaginal septum. Twenty-two silver wire sutures were employed after Sims's method. Union by first intention followed, notwithstanding the failure of the nurse the third day to keep the catheter *in situ*, which allowed several ounces of the urine to accumulate in the bladder. Notwithstanding the decrease in the size of the bladder necessitated by the operation, the patient experiences no difficulty in retaining the urine all night. The operator is not aware that another case of this kind has been previously reported.

THE PRESIDENT asked if it was not possible that some portion of the upper wall of the vagina was drawn upwards and backwards by the bladder, and what was taken to be a continuous wall of the bladder might be a part of the vagina. He had seen a case where there was a large laceration into the bladder, and the opening seemed one cavity with continuous walls, but the flap thrown backwards was post-vaginal, and it was found that this flap could be drawn up and into its position.

PROFESSOR E. C. DUDLEY, in answer to questions, said that the loss of tissue at the base of the bladder from sloughing differs from that by incision. In the latter case the uterus would perhaps be included in the excised tissue, but it is seldom that a slough of the base of the bladder in the vesico-vaginal fistula, however extensive, destroys the connection between the bladder and kidneys. Even if the points through which the uterus penetrate the mucous coat of the bladder be lost, it is yet possible their openings into the bladder may be preserved, because the uterus penetrate the muscular coats nearly an inch from their normal points of opening through the mucous coats of the bladder, and run obliquely between the two coats for a distance of nearly an inch. In this case, as in many cases of loss of entire base of bladder, reported by Emmet, the openings of the ureter were on either side, at the very margins of the fistulous opening. The operation was performed at Morton, Ill., in the presence of Dr. Harris, of that place, and Dr. Mansfield, of Metamora. Dr. Parkes's surmises with reference to the vaginal wall could hardly be correct, because this, together with the anterior wall of the cervix uteri up to the internal os, had sloughed away. His surmise might be correct with reference to certain tissues between the bladder and cervix uteri, which might have retracted and become adherent by inflammation, so as actually to form a portion of the bladder wall. Moreover, there is always a very decided difference in color and appearance between vaginal and bladder tissue, and the tissue in this case was to all appearance like the tissue of the rest of the bladder, and to the touch gave the sensation of a thin wall. Dr. Baker, of Boston, reports a case similar to this, in that he introduced sutures into the bladder tissue, but so close to the cervix uteri as not to draw down any portion of the interior of the bladder, to be used as material in place of the lost vaginal wall.

Dr. W. L. Axford reported

### A CASE OF REMOVAL OF THE ENTIRE LOWER JAW THROUGH THE MOUTH.

Harry T., aged five. Admitted to St. Joseph's Orphan Asylum in November, 1885. It was noticed that his mouth was frequently swollen and sore. Child very much emaciated. In January, 1886, he had measles. Tedious convalescence followed. Came under observation about February 1. Weak and thin. Lower part of face very much swollen. Breath offensive. Symphysis of jaw bare. Could not examine further at this time. Pulse 120 to 130. Put the child on supporting treatment, hoping to get him in condition for an operation. No improvement at the end of two weeks. February 16 the patient was anæsthetized and the mouth explored. Found the jaw on either side stripped of its periosteum back to the masseters. Determined to attempt removal through the mouth, as any culling operation involving the loss of much blood would have been fatal at once. Divided the jaw on either side of the symphysis with bone pliers and thus removed a large portion of the body. Seizing the remaining pieces with sequestrum forceps and making moderate traction, they were easily enucleated by the index finger of the left hand. Not more than a tablespoonful of blood was lost. Patient rallied well. Some reaction on second day. On third day the pulse had dropped to 116, and with exception of a swollen parotid on the left side, the child was in better condition than before the operation; so much so that a recovery was confidently predicted. A severe attack of diarrhœa occurring on the morning of the fourth day was followed by death in thirty-six hours.

DR. ARNOLD P. GILMORE exhibited a patient on whom he had performed an operation for

SYMBLEPHARON OF THE LOWER LID, due to a burn by molten iron, and in which three plastic operations had been unsuccessfully performed. Nine months previously the entire lower lid, from external to internal canthus, was adherent to the eyeball, covering almost the entire cornea. This triangular-shaped tissue was covered by a pale membrane of cicatricial tissue. The operator first detached the lower lid and transplanted the conjunctiva of a rabbit. For six weeks the operation was apparently successful, but after an absence of two months from the city he found the lid was again becoming adherent. Six weeks previously Dr. Gilmore made a thorough dissection, freeing the lid and making a deep cul-de-sac, leaving the upper half of the eyeball covered by mucous membrane and the lower half bare. A semi-circular band of conjunctiva, one-third inch wide, was dissected close to the cornea above, leaving a bridge of tissue at each end. This band was dropped into the cul-de-sac below and carefully stitched to the ball. A semi-circular plate of silver, long enough to fill the space between the external and internal canthi, with two holes at the circumference, one-half inch apart, threaded with silver wire, was dropped into the cul-de-sac to prevent adhesions, and fastened by bringing the wires through upon the face and fastening them by small lead plates and perforated shot. For this operation Dr. Gilmore claimed priority. The object of the operation was neither to improve the appearance of the eye nor to restore vision, but to relieve the irritation of the other eye, by allowing coördinate movements of the two eyes. There was enough clear cornea left to make an artificial pupil in case the patient ever lost his well eye. There was little reaction, and at no time much pus, while the well eye has grown stronger in spite of the presence of the plate.

DR. TILLEY thanked Dr. Gilmore for showing this case, but thought that if the Doctor were to go out of town again for two months, as in the first instance, he would find at the expiration of that time the conditions relatively very much the same as on his return after the first operation. He thought there was little fundamental advantage likely to be associated with the operation, as he thought that in a short time the wire and plate would cause a certain amount of atrophy of the intervening tissue and the plate be forced up out of position, making the operation of no avail. If he was so unfortunate personally as to be placed in a similar position, he would have his eye enucleated.

PROFESSOR E. L. HOLMES thought it unwise to say that a certain thing could not possibly be accomplished, but he had been through the experience of putting in plates, and seeing it done, and never saw one permanently successful. It is different with a very narrow symblepharon in which the globe and eyelid are grown together, where by dissection and transplanting the mucous membrane excellent results may be attained. He thought the plate would irritate the cicatricial tissue and cause it to be very much thickened, and after a few months, or weeks even, when everything is removed, there will be the same tendency to creep over the cornea and make adhesions with a broad union. He thought it absolutely impossible to get an artificial eye to fit. A very small eye might be used and temporarily make it appear that the patient was better off, but that small eye will often irritate and cause the cicatrix to increase.

THE PRESIDENT thought this case one of the same category that is so troublesome to the general surgeon, the improvement of deformities from cicatrices of all kinds, in which relief comes only in the way that Dr. Dudley has applied in gynæcology, after the divided cicatrix has been separated as widely as possible, by drawing together the healthy skin or tissue between the two ends of the divided cicatrix. This method has long been in use in general surgery. So far as his experience went, the application of any foreign body between these divided surfaces has never been followed by success, so far as prevention of contraction goes.

THE PRESIDENT presented an

ENCAPSULATED SARCOMA OF THE THIGH.

It had been in alcohol for some time, and was reduced about one-third in size. It had grown the full extent shown in three months, and was removed from an old lady aged sixty-nine. It was found growing upon the posterior part of the upper portion of the thigh; was a firm, smooth tumor to the touch, and as far as external manipulations determined could not be distinguished positively from other parts of the surrounding tissues. He could not determine whether it was or not attached to the bone, but from external appearances it was diagnosticated to be of a malignant type. The external surface was crossed by a large number of varicose veins. After removal it was shown to be a sarcoma. The interesting point was the rapidity of its growth. He thought it a singular coincidence that about a year previous he had removed a similar tumor from the upper portion of the left arm of an old man of seventy-two years, which had also grown to the full size in three months. Upon exposing the tumor a perfect capsule was reached, and it was easily enucleated from its bed.

